# No differences in FBN1 genotype between men with and without abdominal aortic aneurysm

**DOI:** 10.1186/s12872-023-03068-3

**Published:** 2023-01-20

**Authors:** Ida Åström Malm, Rachel De Basso, Peter Blomstrand

**Affiliations:** 1 Department of Natural Sciences and Biomedicine, School of Health and Welfare, Jönköping University, Jönköping, Sweden; 2 Department of Clinical Physiology, County Hospital Ryhov, Jönköping, Sweden

**Keywords:** Abdominal aortic aneurysm, FBN1, Arterial stiffness

## Abstract

**Background:**

Abdominal aortic aneurysm (AAA) is an aortic enlargement in which the transverse diameter reaches at least 30 mm. Certain risk factors, such as age, male gender, and smoking, are well known; however, less is known about the genetic factors involved. Fibrillin-1 (FBN1) is a protein that coordinates the deposition of elastin fibres in the extracellular matrix and is therefore likely to affect the elastic properties in the aortic wall. Previously studies have found associations between the FBN1-2/3 genotype and arterial stiffness, but how different FBN1 genotypes, AAA, and arterial stiffness are related has been less frequently investigated.

**Aim:**

This study aimed to investigate whether there is a difference in FBN1 genotype between men with and without AAA. A further aim was to study whether the FBN1 genotype affects arterial wall stiffness differently in men with and without AAA.

**Methods:**

Pulse wave velocity and FBN1 genotyping were performed in 229 men (159 with AAA, 70 without AAA). Participants were recruited from ultrasound AAA surveillance programs or ongoing ultrasound screening programs from 2011 to 2016.

**Results:**

The distribution of the FBN1 genotype in the AAA and control groups were as follows: FBN1-2/2: 62% vs. 64%; FBN1-2/3: 8% vs. 14%; and FBN1-2/4: 30% vs. 21%, respectively. Men with AAA and FBN1-2/2 had increased central pulse wave velocity (*p* < 0.005) compared to the control group (those without AAA) with the FBN1-2/2 genotype.

**Conclusion:**

No differences were found with respect to FBN1 genotypes between men with and without AAA. The development of AAA in men does not appear to be linked to a specific FBN1 genotype. Nevertheless, men with FBN1-2/2 and AAA have increased central arterial stiffness compared to men with the same FBN1 genotype but without AAA.

## Background

Abdominal aortic aneurysm (AAA) is defined as an aortic enlargement with a transverse diameter greater than or equal to 30 mm. The prevalence of AAA is approximately 1.5–3% in males aged 65–70 years in Europe [1, 2]. Several risk factors for AAA have been identified, such as age, male gender, and smoking [3, 4]. It is estimated that 75% of all primary cases of AAA are caused by smoking [1, 5]. However, genetic variants may predispose individuals to AAA. The heritability of AAA is greater than 70%: If a first-degree relative has AAA, there is a two-fold higher risk of an individual developing it as well [6, 7]. The development of AAA is characterized by the destruction of elastin, loss of smooth muscle cells, and infiltration of inflammatory cells. Nevertheless, the mechanism underlying aneurysm development also involves interactions between genetic and environmental risk factors. Certain risk factors are well known; however, there is less available information about the genetic factors involved [8]. Meta-analysis of genome-wide association studies for AAA have highlighted several potentially novel mechanisms of AAA pathobiology [9, 10]. However, more knowledge about different genetic markers is needed. Fibrillin 1 (FBN1) is a protein that coordinates the deposition of elastin fibres in the extracellular matrix and is a major constituent of extensible microfibrils. FBN1 is therefore likely to affect the elastic properties in the aortic wall [11]. Different pathogenic variants of the FBN1 gene are associated with Marfan syndrome and lead to a decreased amount of FBN1 available to form microfibrils, which function as a skeleton for elastin deposition and control the direction of growth for elastic fibres [12]. Individuals with Marfan syndrome are at increased risk for developing AAA [13] and show increased aortic stiffness, elevated pulse pressure, and aortic root dilatation [14, 15]. Despite knowledge about the impact of FBN1 mutations on the arterial system, less is known about the effects of different FBN1 genotypes on the abdominal aorta. However, several studies have found an association between the FBN1-2/3 genotype and arterial stiffness [16–19], but how the FBN1 genotype and arterial stiffness in men with AAA are related has been less frequently investigated.

The purpose of the study was to investigate whether there is a difference in FBN1 genotype between men with and without AAA. A further aim was to study whether the FBN1 genotype affects arterial wall stiffness differently in men with and without AAA.

## Methods

### Study population

The study population was recruited from an ongoing ultrasound AAA screening program and a regional ultrasound AAA surveillance program in two neighbouring regions in southern Sweden between 2011 and 2016. A total of 229 men (mean age, 70 ± 3.7 years) were included in the study [159 with AAA and 70 controls (without AAA)]. Subjects with cardiac arrhythmia, severe disability, advanced cancer, or language barriers were excluded from the study. The subjects in the AAA group had AAA with a maximum infrarenal aortic diameter greater than or equal to 30 mm on their latest clinical ultrasound examination. All subjects in the control group had an infrarenal aortic diameter of less than 30 mm at their screening examinations in the prior 5 years [20]. All accepted participants provided written consent to participate in the study. The study was approved by the regional ethical review board in Linköping, Sweden (Dnr 2016/143-32).

### Measurements

Participants were instructed to abstain from alcohol for 12 h and from caffeinated beverages and tobacco for 4 h prior to their examinations, which were performed at either the Department of Clinical Physiology at Linköping University Hospital Linköping, Sweden, or Ryhov County Hospital in Jönköping, Sweden. At the time of the examinations, participants were asked to provide information about their smoking status, cardiovascular diseases, and current medications. Blood pressure was measured with participants in the supine position. Systolic, diastolic, and mean arterial pressures were measured using an oscillometric device (Dinamap model PRO 200 Monitor, Critikon). Pulse wave velocity (PWV) was obtained noninvasively using a SphygmoCor system (Model MM3, AtCor Medical, Sydney, Australia). Ankle systolic blood pressure was measured using a Doppler device. PWV was determined as the time the pulse wave travelled between the carotid and the femoral arteries (central PWV) and between the carotid and radial arteries (peripheral PWV) using a Millar pressure tonometer (Millar, Houston, TX, USA) [20]. Bodyweight was determined without shoes and trousers and rounded to the nearest 0.5 kg; height was measured and rounded to the nearest 0.5 cm.

### Laboratory analyses

Blood samples were collected after an overnight fast in prechilled plastic Vacutainer tubes (Terumo EDTA K-3, Tokyo, Japan). Plasma was prepared by centrifugation at 3000× *g* for 10 min at 4 °C. Blood and plasma were stored at – 70 °C until analysis in the chemistry laboratories at Linköping University and Ryhov County Hospitals, Jönköping. Both laboratories are ISO/IEC 17025 accredited by the Swedish Board for Accreditation and Conformity Assessment.

### FBN1 genotyping

DNA was prepared for polymerase chain reaction (PCR) analysis of the variable tandem nucleotide repeat [(TAAAA)n] in intron 28 of the FBN1 gene on chromosome 15 with forward primer 5′—6FAM CAG AGT ACA TAG AGT GTT TTA GGG AGA—3′ and reverse primer 5′—GTT TCT TCC TGG CTA CCA TCC AAC TCC C—3′. PCR was performed in a volume of 12 μL and consisted of an initial denaturation step at 95 °C for 9 min; 35 cycles of denaturation for 30 s, annealing at 65 °C for 30 s (35 cycles), and extension at 72 °C for 30 s; and a final extension at 72 °C for 30 s. A 1-μl aliquot of the PCR product was diluted with 9 μL of highly deionized formamide (GENESCAN™ 500ROX™ size standard) for electrokinetic injection into a capillary electrophoresis system. The DNA fragments were labelled with the ROX™ fluorophore, which resulted in a single peak per fragment under denaturing conditions. The alleles were then identified by the number of base pairs in each peak (2/2: 162 bp, 162 bp; 2/3: 157 bp, 162 bp; and 2/4: 152 bp, 162 bp).

### Statistics

Comparisons between the AAA and control groups were performed using unpaired Student’s *t* tests, Fisher’s exact tests, or nonparametric means tests (Mann–Whitney U tests). The Kruskal–Wallis test and one-way analysis of variance with post-hoc testing were used to compare differences between genotypes. Differences were considered significant at a *p* value less than 0.05. Continuous variables are expressed as the mean ± standard deviation, and categorical variables are expressed as the number of participants and percentage. Statistical analyses were performed using SPSS for Windows, version 27.0 (IBM Corp., Armonk, NY, USA).

## Results

### Demographic data

Only the three most common FBN1 genotypes (FBN1 2/2, FBN1 2/3, and FBN1 2/4) were included in the analysis, patients with other FBN1 genotypes were excluded from this study. A total of 229 males (159 with AAA and 70 controls) were analysed, and demographic data are shown in Table 1. The mean AAA diameter in the AAA group was 42.1 ± 6.1 mm. The AAA group was significantly older (*p* < 0.001) than the control group. They also had a higher body mass index (*p* < 0.05) and higher C-reactive protein levels (*p* < 0.01) compared to those of the controls. No differences were shown between those with AAA and controls regarding the apolipoprotein B (ApoB)/ApoA ratio or the prevalence of diabetes. The frequencies of hypertension and hyperlipidaemia were higher in males with AAA (*p* < 0.001) than in controls. Males with AAA were also more often treated with antihypertension drugs, including diuretics, calcium channel blockers, and statins, than those without AAA (*p* < 0.001). Further, the AAA group included more current and former smokers than did the controls (*p* < 0.001).

**Table 1:** Characteristics of the study population

	AAA	Control	*p* value
	n = 159	n = 70	
Age, years	70 ± 3.9	68 ± 2.9	< 0.001
BMI, kg/m^2^	28.1 ± 4.3	27.0 ± 3.5	0.046
CRP, mg/L	4.5 ± 6.3	2.8 ± 3.5	0.004
ApoB/ApoA	0.66 ± 1.1	0.73 ± 0.18	0.193
Diabetes, n (%)	12 (7.5)	3 (4.3)	0.823
Hypertension, n (%)	128 (80.5)	27 (38.6)	< 0.001
Hyperlipidaemia, n (%)	102 (64.2)	15 (21.4)	< 0.001
AHT, n (%)	89 (56)	21 (30.0)	< 0.001
ACE inhibitors, n (%)	78 (49.1)	22 (31.4)	0.012
Beta-blockers, n (%)	68 (42.8)	14 (20.0)	< 0.001
Calcium antagonists, n (%)	43 (27.0)	8 (11.4)	0.008
Diuretics, n (%)	34 (21.4)	3 (4.3)	< 0.001
Statins, n (%)	114 (71.7)	17 (24.3)	< 0.001
Aspirin, n (%)	96 (60.4)	12 (17.1)	< 0.001
Smokers, n (%)			
Current	97 (61)	21 (30)	< 0.001
Former	39 (24.5)	5 (7.1)	< 0.001

Values are presented as the mean ± standard deviation for continuous variables and number of participants and percent for categorical variables

*ACE* angiotensin-converting enzyme, *AHT* antihypertensive treatment, *ApoB/ApoA* apolipoprotein B/apolipoprotein A ratio, *BMI* body mass index, *CRP* C-reactive protein

### FBN1 and arterial stiffness

In the AAA group, 99 men had the FBN1-2/2 genotype, 13 had the FBN1-2/3 genotype, and 47 had the FBN1-2/4 genotype (Table 2). The distribution in the control group was 45 men with the FBN1-2/2 genotype, 10 with the FBN1-2/3 genotype, and 15 with the FBN1-2/2 genotype. There was no difference in the distribution of FBN1 genotypes between men with and without AAA, and no difference between the FBN1 genotypes in the AAA group or between the FBN1 genotypes in the control group. However, a difference between the FBN1-2/2 genotype and arterial stiffness was evident in those with AAA and the controls: Men with AAA and FBN1-2/2 had a lower ankle-brachial pressure index (ABPI) (1.08 mmHg vs. 1.17 mmHg, *p* < 0.001), peripheral artery augmentation index normalized to heart rate 75 (per AIx HR75) (82.1% vs. 74.0%, *p* < 0.001) aortic augmentation index normalized to heart rate 75 (AIx HR75) (25.3% vs. 20.4%, *p* < 0.001), central systolic blood pressure (cSBP) (125.4 mmHg vs. 118.2 mmHg, *p* < 0.05), and central PWV (12.4 vs. 11.0, < 0.005) compared to controls with FBN1-2/2. There was no difference in blood pressure and PWV in men with AAA and FBN1-2/3 and controls with FBN1-2/3. In men with AAA and FBN1-2/4 and controls with FBN1-2/4, a difference was shown in peripheral and central AIx HR75 (*p* < 0.01 and *p* < 0.05, respectively), where those with AAA had higher per AIx HR75 and AIx HR75 values. A difference between systolic blood pressure (SBP) and cSBP was found in controls with different FBN1 genotypes. Men with FBN1-2/2 had a lower pressure than did controls with other genotypes [FBN1-2/2 = 127 mmHg, FBN1-2/3 = 138 mmHg, FBN1-2/4 = 133 mmHg; *p* = 0.036 (FBN1-2/2 and FBN1-2/3) and *p* = 0.035 (FBN1-2/2 and FBN1-2/4)].

**Table 2:** Pressure and arterial stiffness according to FBN1 genotype in AAA and controls

	FBN1 2/2		*p* value	FBN1 2/3		*p* value	FBN1 2/4		*p* value
	AAA (n = 99)	Control (n = 45)		AAA (n = 13)	Control (n = 10)		AAA (n = 47)	Control (n = 15)	
SBP (mmHg)	133 ± 17.7	127 ± 12.7	0.058	132 ± 25.4	138 ± 15.1	0.577	132 ± 14.4	133 ± 16.1	0.765
DBP (mmHg)	76 ± 9.1	74 ± 7.9	0.171	74 ± 8.0	78 ± 8.7	0.238	78 ± 7.8	77 ± 6.7	0.758
PPP (mmHg)	57 ± 15.2	54 ± 12.0	0.079	58 ± 21.5	59 ± 12.1	0.525	54 ± 11.8	55 ± 12.0	0.863
MAP (mmHg)	97 ± 11.9	93 ± 9.6	0.054	95 ± 14.9	99 ± 11.1	0.525	97 ± 10.2	95 ± 9.8	0.863
ABPI (mmHg)	1.08 ± 0.17	1.17 ± 0.92	< 0.001	1.18 ± 0.13	1.19 ± 0.10	0.751	1.14 ± 0.12	1.19 ± 0.15	0.512
Per AIx HR75 (%)	82.1 ± 11.6	74.0 ± 12.9	< 0.001	78 ± 14.9	76 ± 5.0	0.713	81 ± 11.2	71 ± 9.4	0.007
AIx HR75 (%)	25.3 ± 6.7	20.4 ± 8.3	< 0.001	23 ± 10.0	23 ± 5.5	0.867	25.0 ± 6.8	19 ± 5.9	0.016
CSBP (mmHg)	125.4 ± 17.3	118.2 ± 13.4	0.019	124 ± 26.1	129 ± 16.4	0.462	124 ± 14.2	120 ± 12.4	0.445
CDBP (mmHg)	77 ± 9.3	75 ± 8.0	0.174	75 ± 7.9	80 ± 8.7	0.229	79 ± 8.2	77 ± 6.7	0.429
CPP (mmHg)	48 ± 14.1	43 ± 12.8	0.054	49 ± 22.2	49 ± 12.0	0.525	45 ± 11.8	43 ± 10.0	0.863
PWVcf (m/s)	12.4 ± 3.0	11.0 ± 2.1	0.004	11.7 ± 3.3	11.3 ± 1.9	0.943	12.4 ± 2.8	12.2 ± 3.4	0.340
PWVcr (m/s)	9.4 ± 1.16	9.0 ± 1.03	0.069	9.5 ± 1.3	9.0 ± 1.0	0.483	9.5 ± 1.3	9.4 ± 0.90	0.882
HR (bpm)	59 ± 8.1	58 ± 8.9	0.492	59 ± 9.2	59 ± 9.6	0.808	62 ± 9.6	60 ± 8.4	0.669

Data are presented as the mean ± standard deviation

*ABPI* ankle-brachial pressure index, *AIx* aortic augmentation index, *CDBP* central diastolic blood pressure, *CPP* central pulse pressure, *CSBP* central systolic blood pressure, *DBP* diastolic blood pressure, *HR* heart rate, *HR75* normalized to heart rate 75, *MAP* mean arterial pressure, *ns* not significant, *Per AIx* peripheral aortic augmentation index, *PPP* peripheral pulse pressure, *PWVcf* carotid-femoral pulse wave velocity, *PWVcr* carotid-radial pulse wave velocity, *SBP* systolic blood pressure

## Discussion

The results of the present study demonstrated that the distribution of FBN1 genotypes in those with AAA was similar to those without AAA. The presence of AAA was not higher in any of the investigated three FBN1 genotypes. However, men with AAA and FBN1-2/2 had increased central arterial stiffness compared to controls with FBN1-2/2. This difference between those with and without AAA was not shown in men with FBN1 genotypes 2/3 or 2/4.

Elastic fibres are important components of all elastic tissues, including large-diameter blood vessels, and the main component of elastic fibres, in addition to elastin, is fibrillin. In elastic arteries, that is, the abdominal aorta, FBN1 is found in all three layers of the arterial wall [21, 22]. FBN1 provides structure to elastic and nonelastic connective tissues via its interactions with elastin and other proteins [11] and plays a vital role in regulating the formation of transforming growth factor-β (TGF-β) complexes in the extracellular matrix. TGF-β signalling controls several processes at the cellular level, such as cellular growth, differentiation, and apoptosis [23]. FBN1 is a glycoprotein with 47 epidermal growth factor (EGF)-like domains, where 43 of these bind calcium. Thus, when the FBN1 gene is mutated, the function of EGF is changed, resulting in insufficient calcium binding [12]. Elastic fibres, found in the aortic wall, are also affected, resulting in decreased elastic properties; the artery will be less compliant and more likely to dilate and develop an aneurysm [24]. Powell et al. showed a strong interaction between FBN1 genotype and blood pressure, which may contribute to the development of aneurysmal disease [16], though we could not show any difference in men with and without AAA according to FBN1 genotype.

In a previous publication on this population, we found a difference between men with and without AAA in terms of central arterial stiffness, measured as PWV [20]. This difference between men with and without AAA in the present study was only shown in men with the FBN1-2/2 genotype. Several studies have found an association between arterial stiffness and FBN1-2/3 in males [16–19], but not in men with FBN1-2/2. Nevertheless, when the controls were compared according to FBN1 genotype, a significant difference in SBP was found, as controls with FBN1-2/3 had higher SBP. The previously demonstrated connection between FBN1-2/3 and arterial function in healthy men [16–19] does not appear to be present in men with AAA. However, FBN1-2/2 was present in over half of men with and without AAA, which may have influenced the result.

It is known that PWV increases with rising blood pressure [25]; thus, it is not surprising that blood pressure differences were observed in those with and without AAA with FBN1-2/2, where men with AAA had a higher SBP. More surprising was the difference in ABPI, where the AAA group had a lower ABPI than the controls. AAA has traditionally been thought to be a focal manifestation of advanced atherosclerosis [26–28]. However, there are differences between atherosclerosis and AAA, as smoking and male gender are much more central risk factors for AAA than for atherosclerosis. Moreover, diabetes is an important risk factor for atherosclerosis but seems to be a negative or neutral risk factor for AAA [29]. Results from recent studies show that peripheral artery disease is negatively correlated with AAA and that atherosclerotic occlusive disease is associated with slower AAA growth [30, 31].

A higher percentage in the AAA group were treated with anti-hypertensive drugs. The use of anti-hypertensive drugs may have an impact on arterial impedance. For example, calcium channel blockers and ACE inhibitors may affect arterial stiffness and reduce PWV [32]. Nevertheless, despite the higher use of these drugs, the men with AAA and FBN1 2/2 had significantly higher PWV compared with controls with FBN1 2/2.

## Limitations

In the present study, the following limitations should be noted. The study population consisted only of men aged 55 to 80 years, with a small AAA. It is a limitation that only men are included in the study, however, the prevalence of AAA is higher in men than in women, women are less than one-fourth as likely as males to have an AAA. The statistical power would be too small if females would be included. Additionally, there was a large difference in the number of participants in the different groups, where FBN1-2/2 had significantly more participants than the other FBN1 groups, in men with and without AAA, which may have impacted the results. Thus, a larger sample size would have been helpful. Moreover, the men with AAA consist of a larger proportion of smokers and former smokers and were more often taking medication. The study was conducted in Sweden, thus most of the participants were white/Caucasian and may not apply to all ethnic population groups. As the study is a cross-sectional study, no direct cause-and-effect associations can be derived.

## Conclusion

In conclusion, this study showed no influence of FBN1 genotype on the prevalence of AAA in men. Consequently, the FBN1 genotype does not seem to be crucial for the development of AAA in men and does not appear to be a useful marker to determine who has a predisposition to develop AAA. However, men with FBN1-2/2 and AAA have increased central arterial stiffness compared to men with the same FBN1 genotype but without AAA. Thus, further studies are needed to fully understand the impact that different FBN1 genotypes have on the mechanical properties of large arteries in men with AAA.

## Data Availability

The datasets generated and analysed during the current study are available in the Zenodo repository, https://zenodo.org/record/7059802#.YxreXHZBxaQ.
